# Specific immune responses after BNT162b2 mRNA vaccination and COVID-19 infection

**DOI:** 10.3389/fimmu.2023.1271353

**Published:** 2023-10-17

**Authors:** Simona Arientová, Kateřina Matúšková, Oldřich Bartoš, Michal Holub, Ondřej Beran

**Affiliations:** ^1^ Department of Infectious Diseases, First Faculty of Medicine, Charles University and Military University Hospital Prague, Prague, Czechia; ^2^ Military Health Institute, Military Medical Agency, Prague, Czechia

**Keywords:** COVID-19; SARS-CoV-2, vaccination, BNT162b2, humoral immunity, T cell immunity

## Abstract

Although vaccines against COVID-19 are effective tools in preventing severe disease, recent studies have shown enhanced protection after vaccine boosters. The aim of our study was to examine the dynamics and duration of both humoral and cellular immune responses following a three-dose regimen of the BNT162b2 mRNA vaccine. In a longitudinal prospective study we enrolled 86 adults who received the BNT162b2 vaccine, 35 unvaccinated individuals with a history of mild COVID-19 and a control group of 30 healthy SARS-CoV-2 seronegative persons. We assessed the SARS-CoV-2-specific T cell responses and IgG production up to 12 months post the third BNT162b2 dose in 24 subjects. The vaccinated group had significantly higher IgG antibody levels after two doses compared to the convalescent group (p<0.001). After the third dose, IgG levels surged beyond those detected after the second dose (p<0.001). Notably, these elevated IgG levels were maintained 12 months post the third dose. After two doses, specific T cell responses were detected in 87.5% of the vaccinated group. Additionally, there was a significant decrease before the third dose. However, post the third dose, specific T cell responses surged and remained stable up to the 12-month period. Our findings indicate that the BNT162b2 vaccine induces potent and enduring humoral and cellular responses, which are notably enhanced by the third dose and remain persistant without a significant decline a year after the booster. Further research is essential to understand the potential need for subsequent boosters.

## Introduction

Coronavirus disease 2019 (COVID-19), caused by severe acute respiratory syndrome coronavirus 2 (SARS-CoV-2), remains a significant public health threat worldwide (1). Vaccines against COVID-19 are highly efficient tools for the prevention of severe disease and death (2). The mRNA vaccine BNT162b2 (Pfizer-BioNTech) contains the spike protein and has demonstrated high safety and efficacy in studies and clinical practice (3, 4). Similar to other viral infections, SARS-CoV-2-specific humoral and cellular immune responses are important for prevention of the infection, viral dissemination and subsequent clearance of the virus (5). Memory immune responses are also critical in protection against reinfections (6).

Current mRNA vaccines induce a robust antibody immune response, which soon begins to decline (7, 8). Several studies have reported an association between the decrease in circulating antibodies and a higher risk of COVID-19 (9). The contribution of specific T-cellular immunity to protection against SARS-CoV-2 infection has also been documented (10, 11). Vaccination with mRNA vaccines induces the development of specific T cells, but their role in long-term protection against the disease is poorly understood (12).

The viral evolution and development of new variants caused decreasing effectiveness of the immunization and led to booster dose strategies. Recent studies have documented better protection against COVID-19 infection after vaccine boosters, but data about the dynamics and duration of immune responses are scarce (13).

Therefore, the aim of our study was to evaluate the dynamics and duration of humoral and cellular responses after 2-dose BNT162b2 mRNA vaccination and after the third (first booster) dose.

## Patients and methods

### Study design and participants

**Figure 1:**
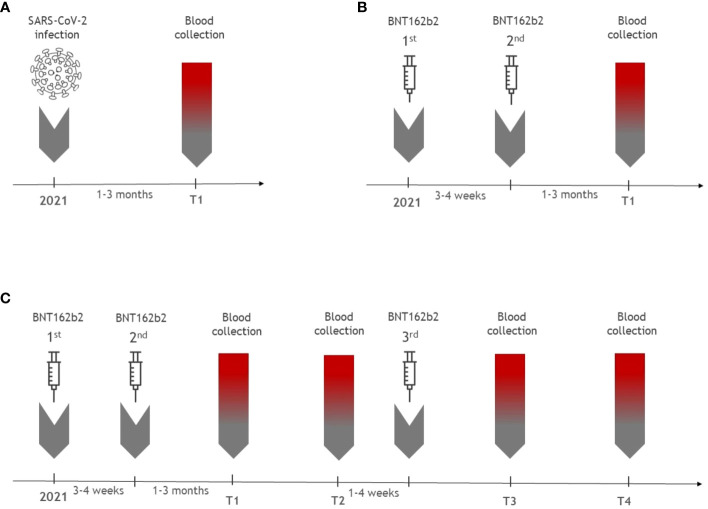
The overview of blood sampling time points (T) in the cohorts. **(A)** illustrates the timing of the blood draw for 35 patients with a history of mild COVID-19. Samples were collected 1-3 months after SARS-CoV-2 infection (T1). **(B)** shows blood sampling of the group of 86 vaccinated individuals post their second vaccine dose. **(C)** provides blood draw schedule for the group of 24 patients who underwent the complete follow-up after receiving all three doses of the mRNA vaccine. Samples were collected 1-3 months after the second dose of the BNT162b2 vaccine (T1), 8-9 months after the second dose (T2), 1 month after the third dose (T3) and 10-12 months after the third dose (T4).

A longitudinal prospective study was conducted in the Vaccination Centre of the Department of Infectious Diseases, Military University Hospital Prague. A group of 86 adults, aged over 18 years, vaccinated with 2 doses of the BNT162b2 vaccine was enrolled between January and March 2021. A group of 35 patients with a history of mild COVID-19 (post-COVID-19) and a control group of 30 healthy SARS-CoV-2 seronegative persons were enrolled between May and June 2021. Demographic parameters of all participants are presented in **Table 1**. A SARS-Co-V-2 infection in the post-COVID group was confirmed by RT-PCR positivity from nasopharyngeal swabs. The individuals had not been vaccinated before the study and their blood samples were taken 1-3 months after their SARS-CoV-2 infection. We analyzed SARS-CoV-2-specific T and B cell immunity in the entire vaccinated group after their second dose and compared it with the post-COVID-19 and control groups. Blood samples for follow-up were taken at these intervals: 8-9 months after the second dose before the third dose (T2), 1 month after the third dose (T3) and 10-12 months after the third dose (T4). The blood draw schedule is depicted in **Figure 1**. Out of the entire vaccinated cohort, 24 participants fully adhered to the study protocol, allowing us to assess the kinetics of both cellular and humoral immune responses at every scheduled time-point. Sampling continued until December 2022. Notably, the main reasons for participant exclusion were SARS-CoV-2 infections after the second vaccine dose and non-adherence to the study protocol. The study was approved by the Ethics Committee of the Military University Hospital Prague. All participants provided informed consent upon enrollment. The study was conducted in accordance with the last version of the Declaration of Helsinki.

**Table 1:** Demographic characteristics of the control group (n=30), the post-COVID-19 group (n=35), the group of all vaccinated persons (n=86) and the group of vaccinated subjects who underwent the complete follow-up after receiving the second dose (D2) and the third dose (D3) of the BNT162b2 vaccine (n=24).

		Controls	Post-COVID-19	Vaccinated	
				All	Follow-up after D2 and D3
**Number of patients**		35	30	86	24
**Female**		19 (54%)	20 (66%)	59 (69%)	19 (79%)
**Male**		16 (46%)	10 (34%)	27 (31%)	5 (21%)
**Age (mean/range)**		48 (25-82)	52 (33-70)	48 (24-79)	49 (26-66)
**Age**	**18-40**	12	5	24	5
**categories**	**41-59**	16	17	48	16
**(years)**	**≥60**	7	7	14	3

### Methods

All peripheral blood samples were collected in VACUETTE® EDTA tubes (Greiner Bio-One, Austria). The fresh samples were processed on the day of collection. Plasma aliquots were immediately frozen at -20 °C and thereafter at -80 °C for subsequent ELISA analyses.

### Analysis of SARS-CoV-2-specific T cell immunity

Spike protein-specific T cell responses were evaluated by the Quan-T-Cell SARS-CoV-2 test and Quan-T-Cell ELISA (Euroimmun^TM^, Luebeck, Germany). The principle of the test is based on the determination of IFN-gamma produced by CD4 and CD8 T cells as well as natural killer cells. The Quan-T-Cell SARS-CoV-2 stimulation tube set consists of three stimulation tubes (BLANK, TUBE, STIM). Fresh human whole blood is incubated in tubes for 24 hours: BLANK: no T cell stimulation, used to determine individual IFN-gamma background, which is subtracted from the following two tubes; TUBE: specific T cell stimulation for SARS-CoV-2 spike protein components of the S1 domain; STIM: nonspecific T cell stimulation to anchored mitogen, used as a positive control. After incubation, all three tubes were centrifuged at 12,000g/10 min at room temperature, and stimulated heparin plasmas were collected in microcentrifuge tubes. Samples were stored in a freezer at -20 °C for a maximum of two months and then measured using a Quan-T-Cell ELISA kit. In accordance with the recommendations from Euroimmun^TM^, values less than 200 mIU/ml were considered negative, while values greater than 200 mIU/ml were considered positive.

### Analysis of SARS-CoV-2-specific humoral immunity

For all participants, plasma samples were stored after blood draw at -80 °C. SARS-CoV-2-specific antibodies were analyzed using Anti-SARS-CoV-2 IgG ELISA test (Euroimmun^TM^, Luebeck, Germany) according to the manufacturer´s instructions. The results were expressed as binding antibody units (BAU/ml). Samples were considered positive when values were ≥ 33.8 BAU/ml.

### Statistical analysis

All statistical analyses were conducted by a certified biostatistician (O.B.) using the R software for statistical computing (R Core Team 2019, Vienna, Austria). We evaluated differences between the study's participant groups based on two primary parameters: sex and age classes. To assess the distribution equality of these parameters across the cohorts, we employed the Fisher's exact test. Data normality was assessed using the Shapiro-Wilk test. To determine any statistically significant differences between defined groups, we used a non-parametric Kruskal-Wallis test followed by pairwise Wilcoxon rank sum tests with corrections for multiple testing (pairwise.wilcox.test). Correlations among individual parameters were evaluated using Pearson's product moment correlation coefficient. In all cases, only p values <0.05 were assumed to be significant.

## Results

The cohort of 86 healthy persons with a mean age of 48 years was vaccinated with two doses of the BNT162b2 vaccine. At T1 after the second dose of the vaccine, anti-SARS-CoV-2 IgG antibody levels and specific T cell responses were compared with the group of 35 patients (mean age of 48 years) with a recent history of SARS-CoV-2 infection (mean interval of 2 months). The results of the comparison of the immunological parameters between the groups are shown in **Supplementary Figure 1**. After the second dose, IgG antibody levels were significantly higher than in the post-COVID-19 group (p<0.001). Specific T cell responses evaluated using the IFN-gamma release assay were not significantly different between the groups. Moreover, a weak correlation was found between humoral and cellular responses (r=0.40, p=0.0003).

**Figure 2:**
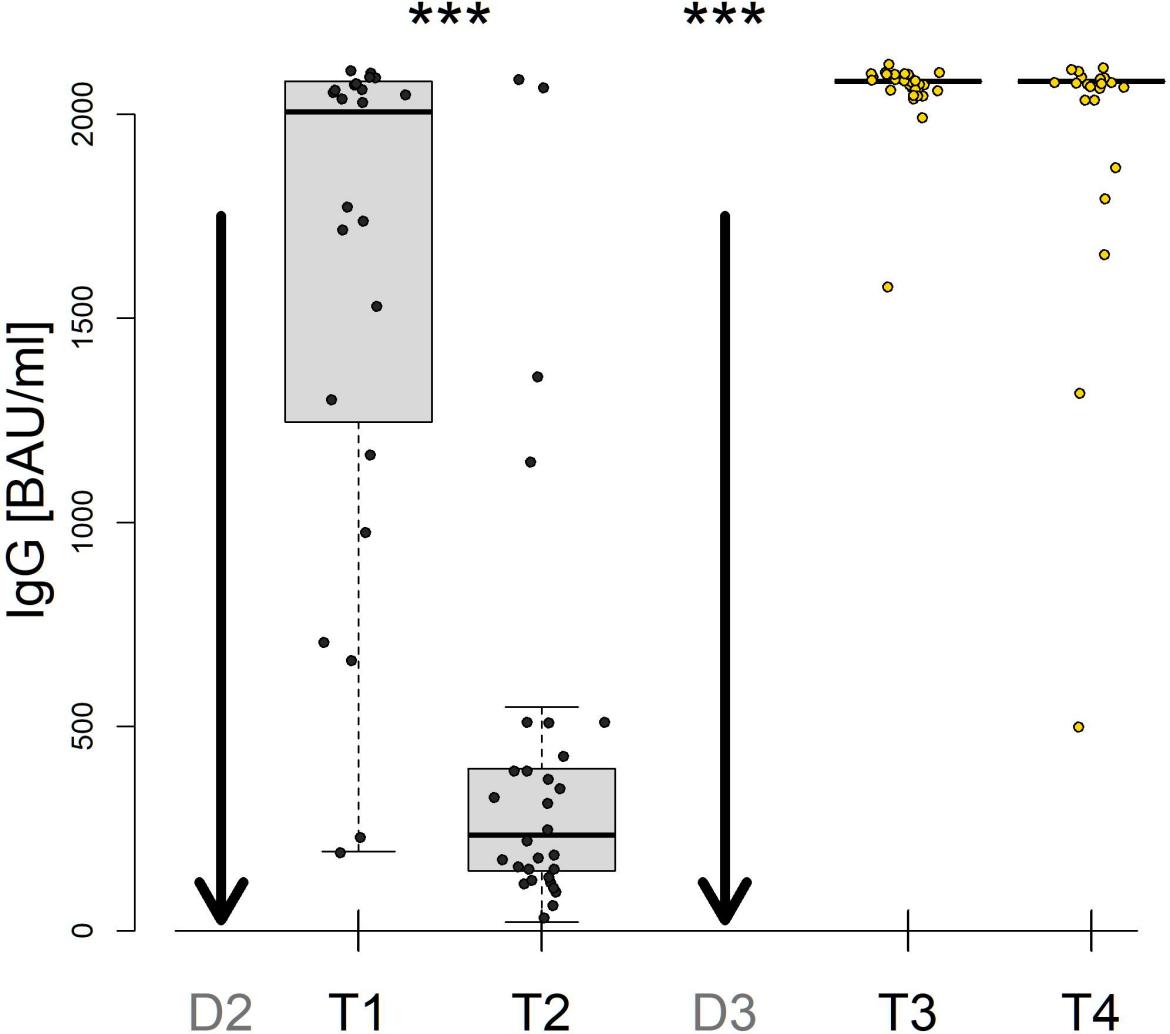
SARS-CoV-2 spike-specific IgG levels after vaccination with the second dose (D2) and the third dose (D3) of the BNT162b2 vaccine. Samples were collected from 24 patients 1-3 months after D2 (T1), 8-9 months after D2 (T2), 1 month after D3 (T3) and 10-12 months after D3 (T4). *** denote P values less than 0.001.

A total of 24 vaccinees provided samples at all time points after the second dose (T1 and T2) and after the third dose (T3 and T4). The IgG levels were positive in all vaccinated participants at all time points (**Supplementary Table 1**). The IgG levels progressively decreased between T1 and T2, but after the first booster dose at T3, IgG titres increased to higher levels than those at T2 (p<0.001, **Figure 2**). Interestingly, only a nonsignificant decrease in IgG levels was observed between T3 and T4.

**Figure 3:**
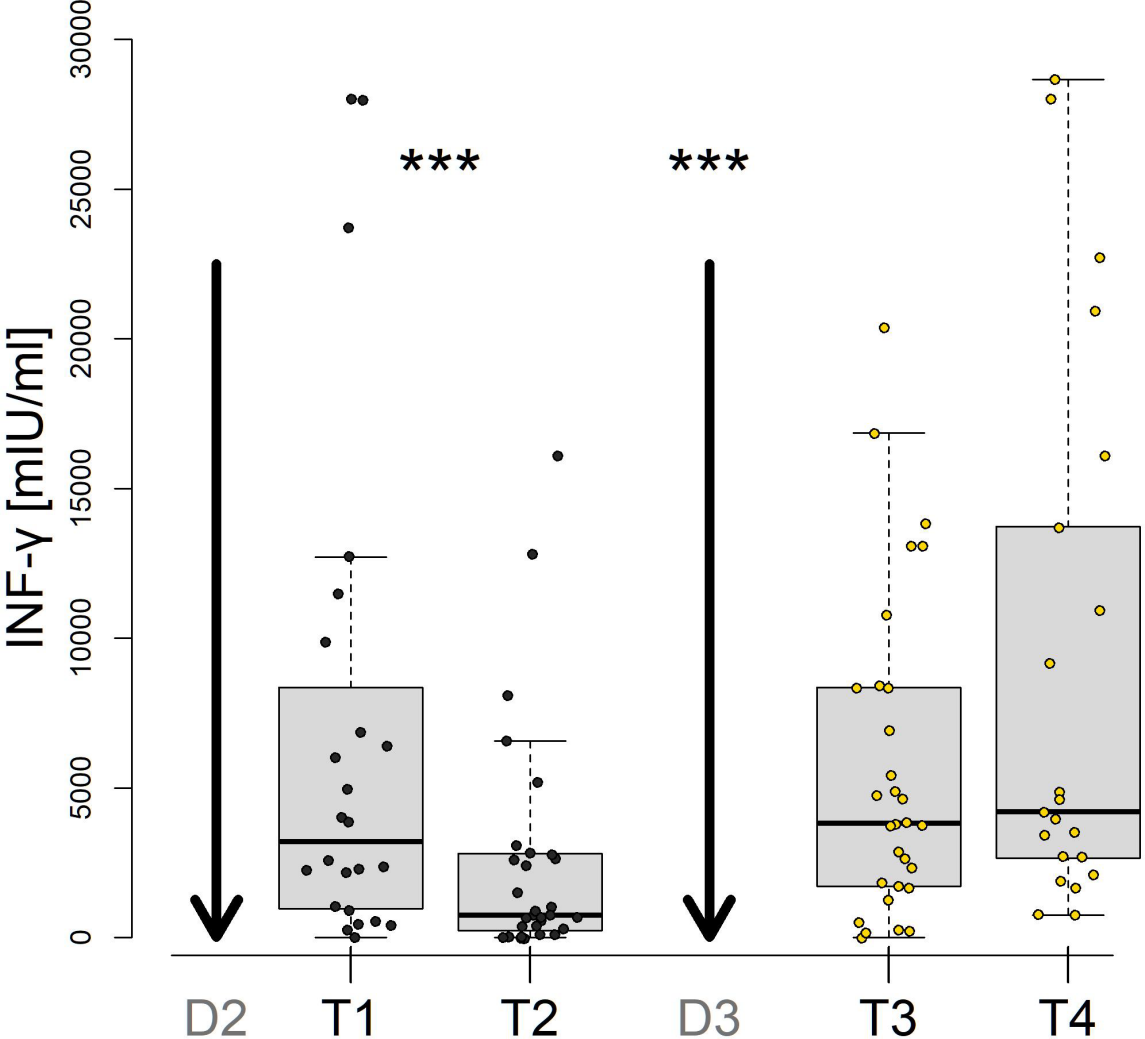
SARS-CoV-2-specific INF-gamma T-cell responses after vaccination with the second dose (D2) and the third dose (D3) of the BNT162b2 vaccine. Samples were collected from 24 patients 1-3 months after D2 (T1), 8-9 months after D2 (T2), 1 month after D3 (T3) and 10-12 months after D3 (T4). *** denote P values less than 0.001.

After two doses, specific T cell responses were detected in 21 vaccinated subjects (87.5%) at T1 and T2 (**Supplementary Table 1**). Additionally, there was a significant decrease before the third dose. However, post the third dose, specific T cell responses surged increased (p<0.001) and remained stable up to the 12 month period (**Figure 3**). Moreover, in 2 out of the 3 subjects with negative results after the second dose, specific T cell responses developed after the third dose (T3 and T4). One patient was persistantly negative for T cell responses throughout the follow-up.

## Discussion

In this study, we evaluated the humoral and cellular immune responses in vaccinated subjects after three doses of the BNT162b2 mRNA vaccine at four time points with a last time point 12 months after the booster dose. The results in this cohort documented a very robust increase in specific IgG levels and a moderate increase in specific T cell responses after the vaccine booster. Both humoral and cellular responses remained stable after the booster up to 12 months of follow-up. Interestingly, postvaccination titres of specific IgG antibodies after the second dose were higher than those in convalescent sera of patients with a mild course of COVID-19. This finding align with a previous study by Hurme et al., which reported elevated IgG levels in healthcare workers who had received two doses of the BNT162b2 vaccine, in comparison to individuals who had recovered from a natural COVID-19 infection (14). Conversely, a study research by Keshavarz et al. indicated that while the BNT162b2 vaccine elicited IgG levels that were lower than those observed in patients hospitalized due to COVID-19, the mRNA-1273 vaccine (Moderna) did not show this trend (15). Such variations can be attributed to multiple factors: timing of measurements, vaccine type, population differences, severity of COVID-19 etc. While B cell responses varied in our study, T cell response levels remained consistent across all groups. Specific IgG levels after immunizations against COVID-19 waned within 5-8 months after the second dose but remained stable after the booster dose. The decrease in humoral immunity after vaccination was shown in previous studies to correlate with age (16). The long-term persistence of high IgG titres after the first booster in our study probably reflects the low mean age of our vaccinated cohort. Moreover, high IgG levels in some persons could be caused by unrecognized exposure to the Omicron variant during 2022. Grikscheit et al. documented that sera from subjects who contracted SARS-CoV-2 Omicron BA.1 after the first or second booster exhibited significantly elevated IgG levels against SARS-CoV-2 Spike-RBD compared to persons vaccinated with a second booster (17). Elevated levels of IgG antibodies after breakthrough infections in previously vaccinated persons were described in other studies (18).

The cellular immune responses, including T cell memory subsets, provide protection against SARS-CoV-2 together with humoral responses (19). Presence of SARS-CoV-2-specific CD4 and CD8 T cells were found to be associated with a decreased severity of COVID-19. Functional memory T cell responses were also detected in vaccinees, and the magnitudes were shown to be comparable to those in convalescent patients. Accordingly, we found similar levels of T cell production of IFN-gamma upon stimulation in vaccinated persons 1-3 months after the second dose and in patients after COVID-19. Similar to the humoral responses, T cell responses waned within the interval after vaccination with the second dose but increased and remained stable after the first booster dose during the follow-up period. Interestingly, three vaccinated persons showed a lack of IFN-gamma production after the second dose with positive IgG levels. Moreover, only one person remained negative for IFN-gamma after the first booster, and the other two subjects converted to moderate positivity of the T cell response.

In conclusion, our findings indicate that the BNT162b2 vaccine induces potent and enduring humoral and cellular responses, which are notably enhanced by the third dose and remain persistant without a significant decline a year after the booster. Further research is essential to understand the potential need for subsequent boosters.

## Data availability statement

The raw data supporting the conclusions of this article will be made available by the authors, without undue reservation.

## Ethics statement

The studies involving humans were approved by Ethics Committee of the Military University Hospital Prague. The studies were conducted in accordance with the local legislation and institutional requirements. The participants provided their written informed consent to participate in this study.

## Author contributions

SA: Data curation, Writing – original draft. KM: Methodology, Data curation, Investigation, Writing – original draft. OBa: Data curation, Methodology, Writing – review & editing. MH: Conceptualization, Funding acquisition, Methodology, Project administration, Supervision, Writing – review & editing. OBe: Data curation, Conceptualization, Formal Analysis, Writing – original draft, Writing – review & editing.

## Supplementary material

Supplementary Figure 1 Comparison of SARS-CoV-2 INF-gamma T cell responses **(A)** and SARS-CoV-2-specific spike-specific B-cell responses **(B)** in patients after a recent SARS-CoV-2 infection (n=35) vs vaccinated persons after two doses of the BNT162b2 vaccine (n=86) and the control group (n=30).
