# Supplementary figures and images for: Specific immune responses after BNT162b2 mRNA vaccination and COVID-19 infection
Source: Front Immunol. 2023 Oct 17;14:1271353. doi: 10.3389/fimmu.2023.1271353 (PMC10619853; doi:10.3389/fimmu.2023.1271353)

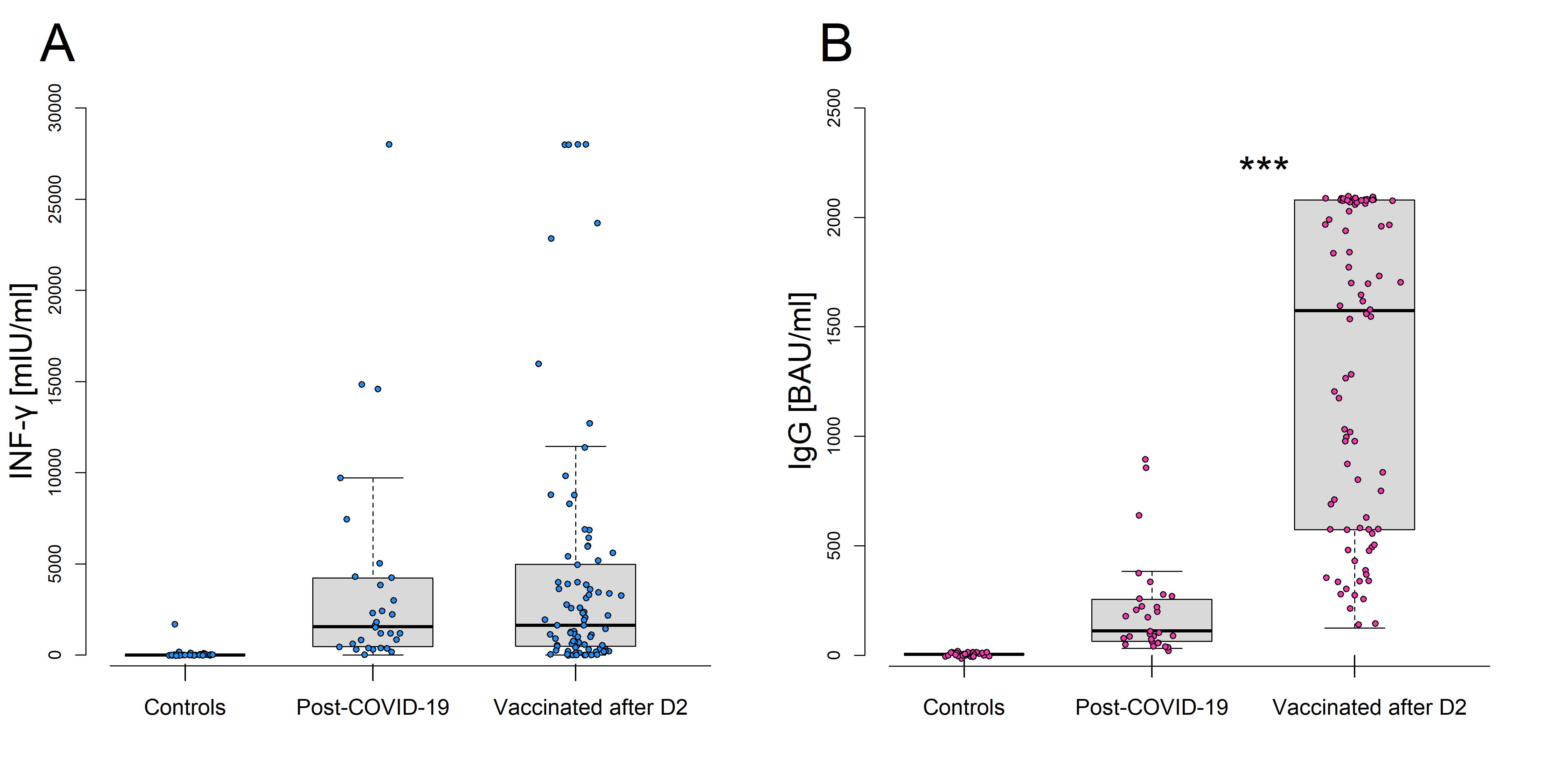

Supplement: Supplementary Figure 1 — Comparison of SARS-CoV-2 INF-gamma T cell responses (A) and SARS-CoV-2-specific spike-specific B-cell responses (B) in patients after a recent SARS-CoV-2 infection (n=35) vs vaccinated persons after two doses of the BNT162b2 vaccine (n=86) and the control group (n=30). [file Image_1.jpeg]
