# Supplementary material for: Specific immune responses after BNT162b2 mRNA vaccination and COVID-19 infection
Source: Front Immunol. 2023 Oct 17;14:1271353. doi: 10.3389/fimmu.2023.1271353 (PMC10619853; doi:10.3389/fimmu.2023.1271353)
Supplement: Supplementary file 2 [file Table_1.docx]

**Supplementary materials**

**Supplementary Table 1** SARS-CoV-2 spike-specific IgG levels and SARS-CoV-2-specific INF-gamma T-cell responses in the control group (n=30), the post-COVID-19 group (n=35), all vaccinated persons (n=86) after the second dose (D2) and the vaccinated group (n=24) during the follow-up after D2 and the third dose (D3) of the BNT162b2 vaccine. Samples were collected 1-3 months after D2 (T1), 8-9 months after D2 (T2), 1 month after D3 (T3) and 10-12 months after D3 (T4).

| **Group** | | | **Anti-SARS-CoV-2-IgG** | | | **IFN-gamma release** | | | |  |
| --- | --- | --- | --- | --- | --- | --- | --- | --- | --- | --- |
|  |  |  | [BAU/ml] **(GM)** | [BAU/ml] **(Median)** | | [mIU/ml] **(Mean ± SD)** | | [mIU/ml] **(Median)** | |  |
| **Controls** | | | 5.4 | 4.8 | | 62.5 ± 288.5 | | 0 | |  |
| **Post-COVID-19** | | | 131.3 | 111 | | 3843 ± 5994.4 | | 1561.1 | |  |
| **Vaccinated** | **All** | | 1061.2 | 1575 | | 4334 ± 6864.4 | | 1632 | |  |
|  | **Follow up after D2** | **T1** | 1135.3 | | 1725 | | 5283.4 ± 7925.1 | | 2239 | |
|  |  | **T2** | 246.4 | | 205.5 | | 2627.9 ± 4192.4 | | 755.4 | |
|  | **Follow up after D3** | **T3** | 2053.1 | | 2080 | | 5726.1 ± 5315.9 | | 3763.9 | |
|  |  | **T4** | 1881.3 | | 2080 | | 8090.5 ± 8819.7 | | 3769.1 | |
| IFN, interferon; n, number of patients; SD, standard deviation; GM, geometric mean; BAU, binding activity units; T, time point; D, dose | | | | | | | | | |  |
